# Lobeline shows protective effects against MPTP-induced dopaminergic neuron death and attenuates behavior deficits in animals

**DOI:** 10.3892/etm.2013.1413

**Published:** 2013-11-18

**Authors:** CHAO-YUE LI, LI-MING ZHAO, XI-WEN SHI, JIA-DONG ZHANG

**Affiliations:** Department of Neurosurgery, Henan Province People’s Hospital, Zhengzhou, Henan 450003, P.R. China

**Keywords:** lobeline, 1-methyl-4-phenyl-1,2,3,6-tetra-hydropyridine, dopamine transporter, substantia nigra, Parkinson’s disease

## Abstract

We previously demonstrated that lobeline effectively inhibited dopamine transporter (DAT)-mediated dopamine (DA) transportation. Therefore, the present study aimed to investigate whether lobeline shows protective effects against neurotoxin-induced cell death *in vivo*. Mice were administered 30 mg/kg 1-methyl-4-phenyl-1,2,3,6-tetra-hydropyridine (MPTP) and treated with 80 mg/kg L-dopa, 10 mg/kg GBR12935 or 1 or 3 mg/kg lobeline, respectively, via injection. Rotarod and swim tests as well as tyrosine hydroxylase (TH) immunohistochemistry were carried out to evaluate the effects of these drugs. Compared with L-DA and GBR12935, lobeline (3 mg/kg administered via intraperitoneal injection) on behavior and dopaminergic neurons. Compared with L-DA and GBR12935, lobeline (3 mg/kg injected subcutaneously) significantly reduced MPTP induced locomotive deficits detected in behavioral tests. In addition, TH immunostaining showed that lobeline (3 mg/kg) markedly decreased the neurotoxin-induced immunoreactivity loss in the substantia nigra and striatum. Lobeline may be useful in the protection of dopaminergic neurons and may alleviate the symptoms of Parkinson’s disease.

## Introduction

Parkinson’s disease (PD) is one of the most common neurodegenerative disorders, and results from the progressive loss of dopamine (DA)-containing neurons in the substantia nigra (SN) and a decrease in DA concentration in the striatum (ST) (1). 1-Methyl-4-phenyl-1,2,3,6-tetra-hydropyridine (MPTP) is a potent neurotoxin that causes selective nigral dopaminergic lesions, resulting in clinical features similar to that of idiopathic PD in human and nonhuman primates. A previous study demonstrated that MPTP-induced dopaminergic cell degeneration was dependent on the presence of dopamine transporter (DAT) (2). Therefore, the blockade of DAT functions may attenuate the accumulation of neurotoxins into dopaminergic neurons and the subsequent neurotoxicity. Several DA uptake inhibitors have been examined in animals and a number of these have demonstrated complete protection against DA depletion in the mouse ST if administered prior to DA-depleting doses of MPTP. The inhibition of DA reuptake may reverse motor deficits in MPTP-treated primates (3,4).

α-Lobeline (lobeline), a lipophilic, nonpyridino, alkaloidal constituent of Indian tobacco, is the predominant alkaloid in a family of structurally-related compounds found in *Lobelia inflata*(5). It has been reported that lobeline may inhibit DA uptake into synaptic vesicles and stimulate the reverse transportation of DA from synaptic vesicles via interactions with the vesicular monoamine transporter 2 (6). The aim of the present study was to investigate whether lobeline exerted neuroprotective effects *in vivo* using MPTP-induced mice models of PD.

## Materials and methods

### Animal preparation and the MPTP-induced model of PD

Male C57BL/6J mice (weight, 30±2 g; age, 4 months) were supplied by the Shanghai Laboratory Animal Center (Shanghai, China). The mice were maintained in cages (n=4 per cage) with access to food and water *ad libitum* and under a 12/12-h light/dark cycle. Prior to the experiments, all mice were trained on the rotarod 3 times a day for 2 weeks. Mice were randomly assigned to six groups (n=10 per group), comprising one group of control mice and five groups of mice treated with MPTP. MPTP-intoxicated mice were administered one subcutaneous (s.c) injection of MPTP-HCl per day (30 mg/kg MPTP per day) for five consecutive days. Lobeline-HCl (1 or 3 mg/kg lobeline, respectively), GBR12935 (10 mg/kg) or vehicle (saline) were administered via s.c injections for 11 consecutive days 30 min prior to MPTP administration. These four groups of mice were known as the lobeline (1 mg/kg)-treated, lobeline (3 mg/kg)-treated, GBR12935 (10 mg/kg)-treated and MPTP-intoxicated (marked as ‘saline’ in figures) groups. In addition, a final group of mice, the L-dopa-treated group, were administered 80 mg/kg L-dopa orally for 11 days as positive controls. Mice in the control group received s.c. injections of saline only. This study was approved by the Zhengzhou University Life-Science Ethics Review Committee (Zhengzhou University People’s Hospital, Zhengzhou, China).

### Rotarod test

Rotarod tests were performed on the 7th, 9th and 11th days. The rotarod testing procedure was a modification of that initially described by Rozas and Labandeira García (7). The overall rod performance (ORP) score for each animal was calculated by the trapezoidal method as the area under the curve in the plot of time-on-the-rod against rotation speed. Time-on-the-rod at each speed was the mean of three values obtained on the three days of testing.

### Swim test

Swim tests were performed on the 12th day in water tubs (length, 40 cm; width, 25 cm and height, 25 cm). The depth of water (27±2ºC) was maintained at 15 cm. The animals were acclimatized for 10 min one day prior to experimentation. The score scales were as follows: 0, hind part sinks with head floating; 1, occasional swimming using hind limbs while floating on one side; 2, occasional floating, mostly swimming; 3, continuous swimming.

### Immunohistochemistry

For tyrosine hydroxylase (TH) immunohistochemistry, mice (n=3 per group) were sacrificed at the end of the swim test and perfusion-fixed with 4% paraformaldehyde in 0.1 M phosphate-buffered saline (PBS) (pH 7.4). Brains were removed, postfixed in the same fixative solution for 12 h and dehydrated in 25% sucrose/PBS solution for 24 h. The entire midbrain and ST were cryosectioned (20 μm for TH) and stored free-floating at 4ºC in a solution of PBS with 0.2% sodium azide. Tissue sections were incubated successively with a rabbit polyclonal anti-TH antibody (1:400), a biotinylated-conjugated polyclonal goat anti-rabbit antibody (1:400) and a horseradish-peroxidase-conjugated avidin/biotin complex. All antibodies were purchased from Vector Laboratories, Inc. (Burlingame, CA, USA). Peroxidase activity was assessed using diaminobenzidine and sections were mounted on glass slides.

The degree of neuronal loss was estimated using a previously described method and an image analysis system (Quantimet color 500, Leica Cambridge Ltd., Cambridge, UK) (8). Neurons were counted on five regularly spaced sections covering the entire SN to estimate the total number of neurons in the SN pars compacta (pc). The optical density (OD) of TH immunoreactivity in the ST was measured using the image analysis system (Quantimet color 500, Leica Cambridge Ltd.).

### Statistical analysis

All values are expressed as the mean ± standard error of the mean. Differences among means were analyzed using one-way or two-way analysis of variance (ANOVA). When ANOVA showed significant differences, pair-wise comparisons between means were tested using Fisher or Newman-Keuls post-hoc tests. In all analyses, the null hypothesis was rejected at a level of 0.05 (two-tailed, unless otherwise stated).

## Results

### Behavioral tests

#### Rotarod test

**Figure 1:**
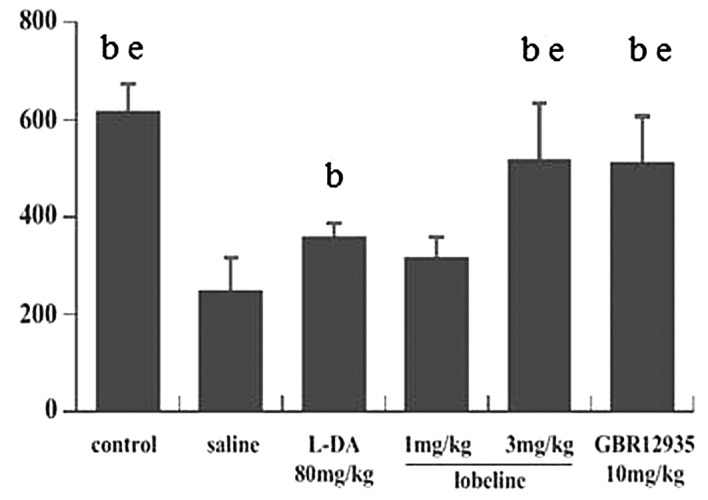
Mean ORP of mice in each group (n=10 per group). Values are presented as the mean ± standard error of the mean of three tests carried out on the 7th, 9th and 11th days. ^b^P<0.05 vs. MPTP-intoxicated mice treated with saline; ^e^P<0.05 vs. the L-DA treatment group. Two-way analysis of variance. MPTP, 1-methyl-4-phenyl-1,2,3,6-tetra-hydropyridine; ORP, overall rod performance.

Mice underwent rotarod testing on the 7th, 9th and 11th days after the first day of MPTP intoxication, and the mean ORP scores were calculated. In all six groups, the mean ORP scores showed no significant changes throughout the experimental period. The mean ORP score of the control group was 617.6±54.86. No significant differences were identified between the lobeline (3 mg/kg)-treated, GBR12935-treated and control groups; however, the mean ORP score of the MPTP-intoxicated group was significantly lower than that of the control group. The mean ORP score of the L-DA-treated group was lower than those of the lobeline (3 mg/kg)-treated and GBR12935-treated groups, although these three groups all showed significantly increased ORP scores compared with that of the MPTP-intoxicated group. However, the mean ORP score of the lobeline (1 mg/kg)-treated group showed no significant difference compared with that of the MPTP-intoxicated group (Fig. 1).

#### Swim test

**Figure 2:**
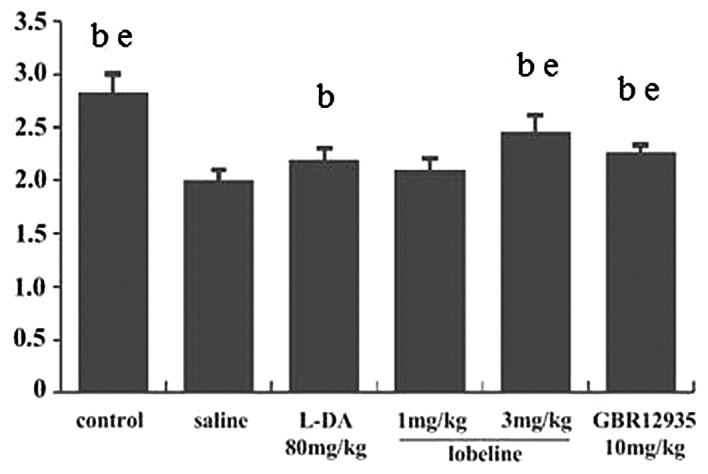
Swim test scores of mice in each group (n=10 per group) on the 12th day. Values are presented as the the mean ± standard error of the mean. ^b^P<0.05 vs. MPTP-intoxicated mice treated with saline; ^e^P<0.05 vs. the L-DA treatment group. Two-way analysis of variance. MPTP, 1-methyl-4-phenyl-1,2,3,6-tetra-hydropyridine.

The swim test was performed once at the end of the experiment, as forced swim tests may result in depression and influence other behavioral tests. The results of the test indicated that lobeline (3 mg/kg) reversed the motor deficit (Fig. 2). No significant differences in swim score were identified between the lobeline (3 mg/kg)-treated, GBR12935-treated and control groups. The swim score in the L-DA-treated group was significantly higher than that of the MPTP-intoxicated group and significantly lower than that of the lobeline (3 mg/kg)-treated group. No significant difference was found between the lobeline (1 mg/kg)-treated and MPTP-intoxicated groups (Fig. 2). The results supported the findings of the rotarod test.

#### Immunohistochemistry

**Figure 3:**
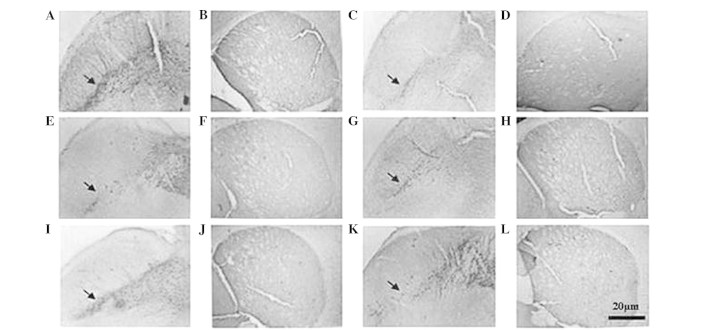
Microphotographs of tyrosine hydroxylase-immunostained sections from the (A,C,E,G,I,K) SN and (B,D,F,H,J,L) ST in (A,B) control mice and MPTP-intoxicated mice treated with (C,D) saline, (E,F) L-DA (80 mg/kg), (G,H) lobeline (1 mg/kg), (I,J) lobeline (3 mg/kg) and (K,L) GBR12935 (10 mg/kg). Note that compared with other treatment options, mice treated with lobeline (3 mg/kg) showed more structural integrity in the SN (indicated by arrows). All treatment options showed more condensation of immunoreactivity in the ST than that of the saline group, with the exception of the L-DA (80 mg/kg) treatment group (magnification, ×400). SN, substantia nigra; ST, striatum; MPTP, 1-methyl-4-phenyl-1,2,3,6-tetra-hydropyridine.

**Figure 4:**
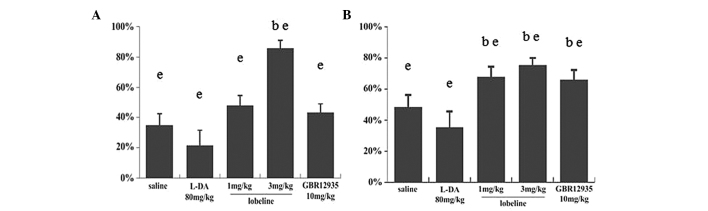
Quantitative analysis of (A) TH-positive cells in the substantia nigra and (B) TH optical density in the striatum in MPTP-intoxicated mice treated with saline or drugs (n=10 per group). The results are shown as a ratio to the corresponding values of the control mice. Data are presented as the mean ± standard error of the mean. ^b^P<0.05 vs. MPTP-intoxicated mice treated with saline; ^e^P<0.05 vs. the control mice. Two-way analysis of variance. TH, tyrosine hydroxylase; MPTP, 1-methyl-4-phenyl-1,2,3,6-tetra-hydropyridine.

TH immunostaining in the mesencephalon enabled dopaminergic neurons in the SN (Fig. 3A, C, E, G, I and K) and ST (Fig. 3B, D, F, H, J and L) to be identified. MPTP intoxication induced a loss of TH immunoreactivity in the SN. At the striatal level, a sharp decrease in TH immunoreactivity was observed in the ST of the MPTP-intoxicated (saline) mice compared with that of the control mice. The decrease in the number of TH-positive neurons was significant in the L-DA-treated group, while minimal in the lobeline (3 mg/kg)-treated group. In addition, the OD of TH immunostaining in the ST and the number of TH-positive neurons in the SN showed less significant reductions in the lobeline (1 mg/kg)-treated and GBR12935-treated mice than that in the MPTP-intoxicated mice (Fig. 4).

## Discussion

The dopaminergic neurotoxin MPTP was introduced in the present study as a paradigm to produce a lesion of dopaminergic neurons in mice. Using rotarod and swim tests, the overall locomotor impairment of mice in different groups was evaluated. MPTP-intoxicated mice showed lower scores than animals in the control group (P<0.05). By contrast, mice that received treatment with lobeline (3 mg/kg) exhibited significantly less impaired mobile abilities than the MPTP-intoxicated mice (P<0.05); however, no significant differences were identified between the MPTP-intoxicated and lobeline (1 mg/kg)-treated groups (P>0.05). In addition, the deficits in locomotor activity were reversed by the administration of L-dopa and GBR12935.

Comparisons of TH immunoreactivity in the SN and ST were performed among the five groups of animals. A marked decrease in the number of TH-positive cells was observed in the MPTP-intoxicated group (~65% compared with the control group). A significant difference was also identified between the lobeline (3 mg/kg)-treated and control groups; however, the decrease was more indiscernible (~15%). Of note, compared with the MPTP-intoxicated group, the decrease in the number of TH-positive neurons appeared more severe in the L-dopa (80 mg/kg)-treated group (~78%, with no statistical significance), while the decreases in the lobeline (1 mg/kg)-treated (~52%) and GBR12935-treated (~56%) groups were less marked.

The results of this study demonstrated that a higher concentration of lobeline (3 mg/kg) may protect cells against MPTP-induced toxicity in dopaminergic systems. L-dopa may have reversed the toxin-induced locomotion deficits; however, it may have accelerated cell death of dopaminergic neurons in the SNpc as reported in a previous study (9). It is of note that GBR12935 was also unable to prevent cells from the disease progress. Unlike lobeline, which serves as a selective inhibitor of DAT-mediated uptake without influence the transporter-mediated release of neurotransmitter, GBR12935 also acts as an inhibitor of DAT-mediated release at low concentrations (5 nM) (10). This may result in an increase in intracellular DA concentration in dopaminergic cells, and increasing endocellular neurotoxins may result in neurotoxicity and neuron death. However, GBR12935 remains an effective agent for symptomatic treatment.

Lobeline is an atypical lipophilic, alkaloidal nicotinic ligand derived from the plant *Lobelia inflata*. Although it shares no obvious structural resemblance to S(−)nicotine and the structure-function associations between nicotine and lobeline do not suggest a common pharmacophore, lobeline has shown mixed agonist- and antagonist-like effects, as well as additional non-nicotinic effects (11). Previous epidemiological studies have reported a significant inverse correlation between cigarette smoking and the incidence of PD (12,13). Furthermore, studies have demonstrated that nicotine may exert neuroprotective effects against excitotoxic insults in neuronal cultures (14,15). Additional studies have supported the findings that nicotine exerts protective effects on dopaminergic systems against MPTP toxicity *in vivo*(16,17). One mechanism underlying this protective action may be its ability to increase the expression of neurotrophic factors that are known to promote survival of dopaminergic neurons (18,19). Thus, the nicotine-like effects may also be accountable for the protective effects of lobeline against MPTP-induced neurotoxicity.

In conclusion, in addition to exerting nicotine-like effects, lobeline, as an inhibitor of DAT, may protect dopaminergic neurons against extracellular toxins, such as MPTP (MPP^+^), by blocking DAT-mediated uptake, and may increase the DA concentration in the synaptic cleft by inhibiting reuptake of DA. Lobeline administration may thus result in the treatment of symptoms and the deceleration of PD progress. Therefore, the combination of several beneficial properties (high blood-brain barrier penetration, DAT inhibition and neurotrophic functions similar to nicotine) suggests that lobeline is a potential preventive or therapeutic agent for the treatment of PD.
